# GLP-1 physiology and pharmacology along the gut-brain axis

**DOI:** 10.1172/JCI194744

**Published:** 2026-01-16

**Authors:** Lisa R. Beutler

**Affiliations:** Department of Medicine, Division of Endocrinology, Metabolism and Molecular Medicine, Northwestern University, Chicago, Illinois, USA.

## Abstract

Historically, antiobesity medications have been modestly effective at best, with side-effect profiles that limit compliance and often preclude the long-term therapy required to maintain weight loss. Recently developed therapies based on analogs of the gut hormone glucagon-like peptide-1 (GLP-1) have transformed the medical management of obesity, leading both to a degree of weight loss that rivals bariatric surgery and a reduction in morbidity and mortality associated with obesity-related complications. GLP-1 receptor agonist (GLP-1RA) therapies were developed to mimic the peripheral effects of GLP-1, but it is now well established that their efficacy in the treatment of obesity depends on reducing energy intake through their action in the central nervous system (CNS). Recent data indicate that the aversive gastrointestinal side effects of GLP-1RAs are also CNS mediated. Although a complete understanding of the neural circuits underlying GLP-1RA–induced weight loss remains elusive, a great deal has been learned in recent years. This Review summarizes proposed gut-brain and central mechanisms through which GLP-1 and its synthetic analogs regulate food intake and bodyweight.

## Introduction

Food intake necessitates a complex organismal response to maintain homeostasis across multiple time scales. Rapid communication between the gut and the brain about recently consumed nutrients regulates metabolism and feeding behavior, both of which are critical for bodyweight maintenance (1). This occurs via multiple mechanisms, including the nutrient-mediated release of a repertoire of peptide hormones from a sparse population of enteroendocrine cells (EECs) within the gastrointestinal (GI) epithelium (2). These signals can act peripherally to impact physiology and metabolism directly. They also may alter neural activity either through direct humoral action in the brain or via activation of local peripheral sensory neurons that densely innervate the GI tract. Neural effects of gut hormones can thus impact both systemic physiology and behavior.

The role of gut hormone signaling in the nervous system is enormously complex, and the physiologic relevance of gut hormones in the regulation of food intake and bodyweight remains controversial. However, when administered systemically at pharmacologic doses, many of these peptides have potent anorexigenic effects. Due to the clinical success of glucagon-like peptide-1 receptor agonists (GLP-1RAs), and to the proliferation of modern neuroscience and genetic approaches (3–6), the mechanisms underlying GLP-1–induced appetite and bodyweight regulation have been studied intensely for over a decade.

Several years after the discovery of GLP-1, and the description of its incretin effect (7–9), multiple groups reported that intracerebroventricular GLP-1 administration suppresses appetite in rodents (10–12), and that 6 weeks of subcutaneous GLP-1 infusion induces modest (approximately 2%) weight loss in humans (13). Surprisingly, despite the clear anorectic effects of pharmacologically dosed GLP-1, GLP-1R–knockout mice exhibited normal feeding behavior and bodyweight on a chow diet and were protected from high-fat diet–induced obesity (11, 14–16). Concomitantly, even the earliest human studies and clinical trials revealed that pharmacologic dosing of GLP-1 resulted in GI side effects, including nausea and vomiting (17). These and other foundational studies generated questions that remain active areas of investigation in the incretin and obesity fields. Central among these are efforts to disentangle the physiologic versus pharmacologic roles of GLP-1 in food intake and energy balance, and to determine which GLP-1R–expressing cells are critical for both the beneficial and on-target adverse effects of GLP-1RA therapy. This Review focuses on recent advances in our understanding of these complex processes and outlines remaining knowledge gaps that must be filled to enable the development of next-generation gut hormone–derived antiobesity therapies with improved efficacy and enhanced tolerability. Specifically, it delineates how GLP-1 signaling in the peripheral nervous system and the central nervous system (CNS) impacts food intake and energy homeostasis. The roles of endogenous GLP-1 signaling and the pharmacologic effects of GLP-1–based therapies are considered separately.

## Basic anatomy of the GLP-1 system

GLP-1, one of several products of the preproglucagon gene, is expressed peripherally in a subset of EECs known as L cells located largely in the distal small intestine and colon (18), although some GLP-1–releasing EECs are located in the proximal small intestine (19, 20). These proximal GLP-1–releasing EECs may account for the rapid rise in circulating GLP-1 following food intake (21–23), though a neuroendocrine feedback loop has also been proposed to account for this (24, 25). GLP-1 is expressed at much lower levels in pancreatic islet α cells, and this pool of peripheral GLP-1 may partially mediate GLP-1’s incretin effect (26–29). GLP-1 is also expressed in the brain, primarily in a subset of neurons in the solitary tract nucleus (NTS) in the brainstem, a critical hub for integrating peripheral signals, including nutritional stimuli from the gut (30–33). GLP-1 has also been detected in the olfactory bulb, although its function there has been minimally explored (33, 34).

GLP-1R is expressed broadly across the gut-brain axis. Conditional knockout and rescue studies indicate that GLP-1R in pancreatic β cells, but not in the brain or peripheral nervous system, is largely responsible for both the physiologic incretin effect of GLP-1 and the glucose-lowering effect of GLP-1RAs (35–37). Of note, although baseline glucose tolerance and GLP-1RA–mediated improvement in glucose tolerance are intact in mice lacking GLP-1R on vagal afferent neurons (37), pharmacologic, GLP-1R knockdown, and vagotomy studies in rats and humans variably implicate vagal afferent GLP-1R signaling in glucose tolerance, the incretin effect, and the glycemic response to GLP-1RAs (38–44). These findings warrant additional investigation. In addition, GLP-1RA delivery directly to the hypothalamus improves glucose tolerance (45, 46), at least in part through suppression of hepatic glucose production, suggesting that the antihyperglycemic effect of these drugs may go beyond recapitulating the endogenous incretin effect. GLP-1 also improves glycemia by suppressing glucagon release through mechanisms that are incompletely understood but appear to be mediated largely through intrapancreatic mechanisms. Taken together, the physiologic and pharmacologic mechanisms of GLP-1R agonism with respect to glucose homeostasis are partially overlapping and at least largely peripherally mediated. The glucoregulatory effects of GLP-1 and GLP-1RAs have been reviewed elsewhere and will not be considered further here (47, 48).

In the peripheral nervous system, GLP-1R is expressed on a large population of stomach-innervating vagal afferent neurons (VANs) critical for detecting GI stretch and transmitting this information to the NTS (49, 50). GLP-1R is also expressed on hepatic portal vein–innervating VANs, and at very low levels on small intestine–innervating fibers (42, 49, 50). GLP-1R signaling in VANs, whose cell bodies reside in the nodose ganglia, may play a limited role in glucose homeostasis as noted above, and are required for slowed gastric emptying induced by native GLP-1 and GLP-1RAs (42, 51, 52). In addition, a recent report described a population of GLP-1R–expressing enteric neurons within the myenteric plexus, known as intestinofugal neurons, that promote stomach distension and anorexia via a spinal afferent circuit (53).

GLP-1R expression in the CNS is clearly required for GLP-1RA–induced weight loss (37). However, GLP-1R is expressed in numerous neural populations distributed throughout the CNS (54), and functional mapping of the role of each of these in maintaining energy balance, in effecting GLP-1RA–induced weight loss, and in inducing GLP-1RA side effects remains the subject of intense study. GLP-1R is expressed in a subpopulation of NTS neurons. Interestingly, the peripheral and central neural GLP-1 systems are at least largely disconnected. NTS GLP-1–expressing neurons do not receive inputs from GLP-1R–expressing VANs; rather, mechanosensitive oxytocin receptor–expressing VANs are the major vagal population that projects to NTS GLP-1 neurons (55). Furthermore, NTS GLP-1–expressing neurons themselves do not express GLP-1R or respond to GLP-1RA (56–58). In addition to the NTS, GLP-1R is also expressed in multiple brain regions, with roles in regulating food intake and energy balance, many of which are established projection targets of NTS GLP-1 neurons (31–33, 59). These include other parts of the dorsal vagal complex (DVC), specifically the area postrema (AP) and dorsal motor nucleus of the vagus (DMV); multiple hypothalamic nuclei, including the arcuate nucleus (ARC), paraventricular nucleus (PVH), lateral hypothalamus (LH), and dorsomedial hypothalamus (DMH); and regions involved in motivated behavior and reward, including the ventral tegmental area (VTA), nucleus accumbens (NAc), and lateral septum (LS), which have an increasingly appreciated role in palatable food intake and the pathophysiology of obesity (60–64).

Of note, several of the central populations of GLP-1R–expressing neurons implicated in feeding and energy balance are in or adjacent to circumventricular regions that lack a blood-brain barrier (BBB) and thus can sense peripherally circulating signals and drugs that do not freely cross the BBB. The ARC houses several neural populations that are critical for regulating appetite and energy balance, including hunger-promoting agouti-related peptide–expressing (AgRP-expressing) neurons (65–69), satiety-inducing proopiomelanocortin-expressing (POMC-expressing) neurons (70, 71), and multiple other populations of appetite- and metabolism-regulating neurons (72–75). Within this region, GLP-1R is expressed in POMC neurons (37, 76, 77) and in a recently described population of thyrotropin-releasing hormone–expressing (TRH-expressing) neurons that send inhibitory projections to AgRP neurons (78). The AP is a circumventricular region in the caudal brainstem that is known for sensing circulating factors, such as signals of nutritional state and toxins, and orchestrating appropriate behavioral and autonomic responses, including vomiting in emetic species (79, 80). The adjacent NTS, described above, is an interoceptive hub and houses multiple anorexigenic neural populations (81, 82). Thus, while acutely administered GLP-1RAs, including liraglutide and semaglutide, do not access most deep brain regions, their actions in and near circumventricular areas are critical for their efficacy as antiobesity medications (83, 84). Moreover, clinically used GLP-1RAs have access to some nuclei farther from circumventricular organs, including the PVH and DMH, at steady state (83).

## Endogenous GLP-1 effects on feeding and energy balance

### Peripheral GLP-1 signaling.

Multiple lines of evidence refute a role for peripherally produced GLP-1 in long-term energy balance, although it may play a limited role in regulating feeding structure. As noted above, global GLP-1R–knockout mice have normal bodyweight, normal food intake on a standard chow diet, and are protected from, rather than more susceptible to, high-fat diet–induced obesity (11, 14–16). In rats, lentiviral knockdown of GLP-1R in VANs had no effect on bodyweight or total daily food intake, although knockdown rats ate larger meals, with a compensatory reduction in meal number (42). Perhaps the most compelling argument that peripheral GLP-1 does not contribute to energy balance is that deleting the preproglucagon gene from the GI tract modestly impaired oral glucose tolerance and increased gastric emptying but had no effect on food intake or bodyweight despite almost completely eliminating circulating GLP-1 (85). In agreement, the GLP-1R antagonist exendin-(9-39) (Ex-9) delivered via microinfusion to the ileum failed to augment food intake (53).

It is possible that the lack of bodyweight phenotype in some genetic models is related to developmental compensation or the redundant effects of multiple anorectic gut hormones on food intake. Antagonist studies have addressed the possible confound of compensation. In addition to local ileal delivery of Ex-9 having no effect on food intake, multiple studies using various peripheral administration routes, including intraperitoneal, jugular venous, and inferior vena cava infusions, showed no effect of GLP-1R antagonism on food intake (86, 87). One group showed that intraperitoneal Ex-9 increased light-cycle feeding and blunted the anorectic effect of a nutrient preload in rats (88). However, it is possible that these effects of systemically delivered Ex-9 were mediated by blockade of central GLP-1R in circumventricular regions. GLP-1R antagonism in humans did not increase caloric intake following a standardized meal or glucose preload (89, 90). In agreement with behavioral studies, GLP-1R antagonism also did not block GI-nutrient-mediated AgRP neuron inhibition in vivo (91–93).

These studies all assessed the acute effects of GLP-1R antagonism. The effects of chronic GLP-1R antagonism are mixed. In agreement with the finding that GLP-1R–knockout mice are protected from diet-induced obesity, one group showed that prolonged GLP-1R antagonism using a blocking antibody modestly attenuated weight gain in mice on a high-fat diet (94). However, others showed that daily administration of a peptide antagonist for one week enhanced weight gain and food intake in diet-induced obese mice (95). A small, two-week clinical trial evaluating GLP-1R antagonism for the treatment of postbariatric hypoglycemia did not change bodyweight in participants, although interpretation of bodyweight changes in this population is challenging (96). Taken together, these studies show that peripheral GLP-1 signaling is not an essential physiologic regulator of bodyweight but may modestly alter meal structure by promoting satiation under certain circumstances. To the extent that this occurs, it may require GLP-1R signaling in VANs (42). The seemingly paradoxical antiobesity effect of both GLP-1R knockout and possibly of chronic GLP-1R blockade are poorly understood. They are likely related to complex downstream metabolic dysregulation induced by the disruption of incretin signaling rather than a proobesity effect of GLP-1 per se (94).

### Central GLP-1 signaling.

More evidence supports a role for central than peripheral GLP-1 signaling in feeding and bodyweight regulation. Chronic delivery of Ex-9 to the lateral ventricle in rats increased bodyweight in chow- and high-fat diet–fed animals (97). Ex-9 delivered to the NTS increased 24-hour food intake and reduced gastric distension–induced appetite suppression (98, 99), while virus-mediated GLP-1R knockdown in the NTS increased food intake and motivation for palatable rewards in rats but did not markedly increase bodyweight after 16 days (100). Chronic fourth ventricle GLP-1R antagonism also attenuated weight loss and increased food intake in a rat model of cancer cachexia (101).

Because circulating GLP-1 is rapidly degraded by dipeptidyl peptidase IV (DPPIV), it is believed to act largely in a paracrine or local rather than hormonal fashion, and it is unclear whether peripheral GLP-1 directly activates brain GLP-1R under physiologic conditions (102, 103). Therefore, it is likely that the effect of central GLP-1R blockade on bodyweight is due to disrupting the action of CNS-derived rather than peripheral GLP-1. Supporting this notion, lentiviral knockdown of the preproglucagon gene in the NTS increased food intake and exacerbated obesity in rats on a high-fat diet and reduced anorexia in rats with cancer cachexia (97, 101). In agreement with behavioral findings, food intake activated NTS GLP-1–releasing neurons with a response magnitude proportional to meal size (104–106). Moreover, optogenetic or chemogenetic stimulation of NTS GLP-1 neurons suppressed food intake without inducing aversion (55, 104, 107). While this artificial neural manipulation alters more than just GLP-1 release and is not necessarily reflective of physiologic function, it is consistent with a role for NTS GLP-1 in satiation and supports the idea that this is not due to malaise or broader behavioral deficits.

Other studies have failed to demonstrate a role for CNS GLP-1 in long-term energy balance. Most, but not all, of these studies support a role for NTS GLP-1 neurons in normal feeding architecture. CNS-restricted GLP-1R–knockout mice have normal bodyweight and food intake (37, 52). Diphtheria toxin–mediated ablation of NTS GLP-1 neurons had no effect on ad libitum food intake or bodyweight in chow-fed mice, although it increased chow intake following a caloric preload and augmented fasting-induced hyperphagia (55, 108). Similarly, chemogenetic inhibition of NTS GLP-1 neurons increased fast refeeding and intake of a palatable liquid (Ensure) in fed mice but did not impact ad libitum chow intake. Finally, unlike the prior observation in rats (97), virus-mediated deletion of the preproglucagon gene in the NTS in mice did not alter bodyweight or total food intake, but it prevented feeding suppression during chemogenetic stimulation of NTS GLP-1 neurons (109).

GLP-1 signaling in multiple projection targets of NTS GLP-1 neurons has also been studied and the results largely reinforce a role for central GLP-1 in the regulation of food intake and possibly energy balance. GLP-1R neuron ablation in the DMH caused obesity and increased food intake in rats (110). Additional studies confirmed that virus-mediated GLP-1R knockdown in rats and mice increased bodyweight, but found this occurred through decreased energy expenditure, not increased food intake (111, 112). Thus, it appears that GLP-1R–expressing neurons in the DMH are important for bodyweight regulation, but the exact mechanisms remain unclear. Germline deletion of GLP-1R in the PVH did not impact bodyweight in chow or high-fat diet–fed mice (45). However, virus-mediated knockout of GLP-1R or chronic GLP-1R antagonism in the PVH of adult mice and rats led to weight gain (84, 113), and chemogenetic inhibition of PVH GLP-1R neurons in mice enhanced food intake in sated animals (114). Thus, the negative results in the earlier knockout study may be due to developmental compensation. Virus-mediated GLP-1R knockdown in the LH in rats led to weight gain and increased food intake (115). Ex-9 in the VTA or NAc acutely increased intake of palatable foods in rats (61), although the effects of chronic antagonism or selective GLP-1R knockout in these regions are unknown. Finally, Ex-9 in the LS modestly enhanced acute sucrose intake and increased operant responding for food rewards in rats and mice (116, 117), and chemogenetically inhibiting GLP-1R–expressing neurons in the LS increased food intake in mice possibly through an inhibitory projection to the LH (118). Taken together, these studies show that while central GLP-1 is not critical for long-term energy balance in all circumstances, it is important for controlling food intake and likely regulates bodyweight in some contexts.

**Figure 1:**
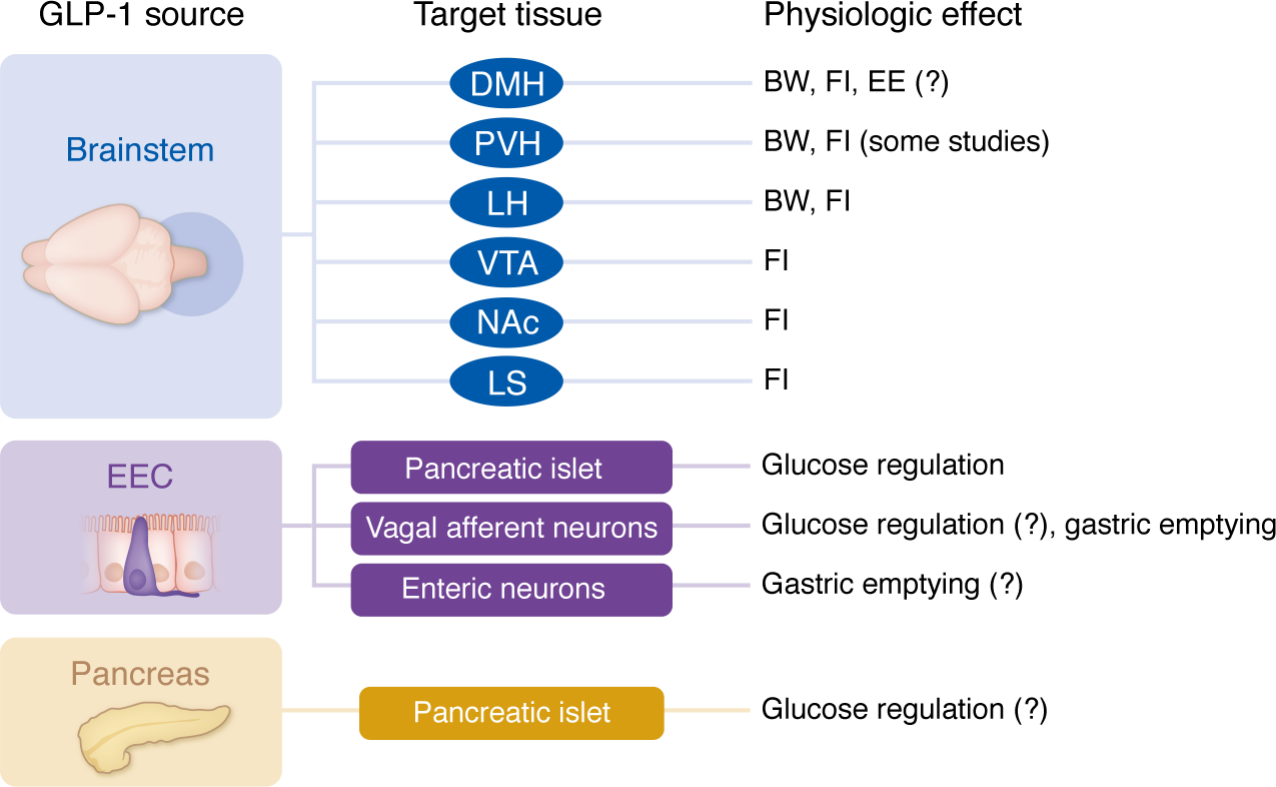
Overview of the physiologic sources, targets, and proposed function of GLP-1 discussed in this Review. GLP-1 is secreted from neurons in the solitary tract nucleus of the brainstem, from enteroendocrine L cells, and from pancreatic α cells. Each source acts on different target tissues, with a range of physiological effects. BW, bodyweight; FI, food intake; EE, energy expenditure; remaining abbreviations are as described in the text.

A schematic depicting a simplified overview of the sources, targets, and functions of endogenous GLP-1 is shown in Figure 1.

## GLP-1 pharmacology and the gut-brain axis

### Anorectic effects.

The effects of endogenous GLP-1 on food intake and bodyweight pale in comparison with the impact of GLP-1 pharmacology. The clinically used GLP-1RAs are structurally modified GLP-1 analogs with dramatically increased half-lives relative to the native peptide (119). This has enabled the remarkable clinical efficacy of these medications, particularly for weight loss. The most recently approved GLP-1RA, semaglutide, which has a half-life of approximately 7 days enabling weekly dosing, has revolutionized the medical management of obesity. There is a rich pipeline of GLP-1–based therapies with potentially greater efficacy in development (120, 121).

GLP-1RAs induce weight loss through action in the CNS. GLP-1R knockout in CNS neurons eliminated dulaglutide- and liraglutide-induced weight loss in obese mice (37, 52). GLP-1R in glutamatergic but not GABAergic neurons was required for this effect (122). Ablating NTS GLP-1 neurons had no effect on liraglutide- or semaglutide-induced feeding suppression, consistent with the fact that these neurons do not themselves express GLP-1R (55–57). By contrast, GLP-1R–expressing neurons in the DVC appear to be a critical site of action for GLP-1RA–induced anorexia and weight loss. Chronic activation of GLP-1R–expressing DVC neurons prevented weight gain in high-fat diet–fed mice, and genetically ablating GLP-1R–expressing neurons in the DVC, but not the ARC or nodose ganglia, prevented semaglutide-induced weight loss in diet-induced obese mice (123). Neither NTS nor AP GLP-1R knockout attenuated acute GLP-1RA–induced feeding suppression in mice, suggesting that GLP-1RA action on either of these populations is sufficient for the acute anorectic effects of these agents (123). However, selective NTS GLP-1R knockout attenuated chronic liraglutide-induced weight loss, while AP lesioning did not (84, 124). These findings suggest that within the DVC, GLP-1Rs in the NTS are key mediators of GLP-1RA efficacy.

Most but not all studies suggest that the ARC is not a critical site of action for GLP-1RA–induced weight loss. Caspase-mediated ablation of ARC GLP-1R–expressing neurons did not block semaglutide-induced weight loss (123), and CRISPR-mediated GLP-1R knockout in the ARC did not affect acute liraglutide-induced anorexia (125). By contrast, chronic Ex-9 delivery to the ARC markedly attenuated liraglutide-induced weight loss (84) and chemogenetic stimulation of all GLP-1R–expressing neurons in the ARC reduced food intake in mice (59). Within the ARC, GLP-1R is expressed in a subset of POMC neurons that are directly activated by GLP-1RAs (76, 77). GLP-1R is also expressed in a recently characterized population of GABAergic TRH-expressing neurons that project locally to inhibit AgRP neurons (78). This finding agrees with multiple studies showing that GLP-1RAs indirectly inhibit AgRP neurons both in slice preparations and in vivo (76, 84, 93). Chemogenetic stimulation of GLP-1R–expressing POMC neurons reduced food intake in mice (126), while GLP-1R knockout in POMC neurons did not attenuate the acute anorectic effect of Ex-4, although it is possible that developmental compensation may account for the lack of effect in this model (45). Optogenetic stimulation of GLP-1R–expressing TRH-positive neurons in the ARC also suppressed feeding via AgRP neuron inhibition, and AgRP neuron stimulation partially overcame Ex-4 or TRH-neuron stimulation–induced anorexia (78, 93). Taken together, these results show that while it is unlikely that direct action in the ARC is the major mechanism for GLP-1RA–induced weight loss, the contributions of genetically distinct subsets of GLP-1R–expressing neurons in this region have not been fully explored. It is also possible that ARC neurons that do not express GLP-1R but do regulate feeding behavior, including AgRP neurons, may play a role in GLP-1RA–induced weight loss through indirect downstream circuit effects.

Other hypothalamic nuclei involved in feeding regulation and energy homeostasis are also impacted by GLP-1RA administration. Liraglutide was shown to potentiate food-induced activation of GLP-1R–expressing neurons in the DMH. These neurons directly inhibit AgRP neurons in the ARC to suppress feeding (127). This study also showed that virus-mediated knockdown of GLP-1R in the DMH modestly attenuated 24-hour liraglutide–induced feeding suppression, but it is not clear whether this circuit is necessary for the chronic weight-reducing effects of GLP-1RAs. Within the DMH, GLP-1R in neurons that coexpress the leptin receptor are sufficient to mediate liraglutide-induced feeding suppression but not weight loss (128); however, whether GLP-1R–positive, leptin receptor–negative neurons in the DMH are critical for the chronic effects of GLP-1RA treatment has not been examined.

By contrast, while GLP-1R antagonism in the PVH increased bodyweight in chow-fed animals, neither antagonism nor GLP-1R knockdown in this region impacted the acute or chronic effects of liraglutide on feeding and bodyweight (84, 125). Moreover, chemogenetic inhibition of PVH GLP-1R neurons enhanced food intake, but did not attenuate acute liraglutide-induced anorexia (114). Together, these data suggest that the substrates for endogenous GLP-1 feeding regulation and the pharmacologic effects of GLP-1RA are distinct.

The brainstem and hypothalamus have dominated studies aimed at dissecting GLP-1RA weight loss mechanisms, and less is known about the role of GLP-1R in other areas of the brain. A recent study showed that viral CRISPR-mediated knockdown of GLP-1R in the LS slightly attenuated acute liraglutide-induced feeding suppression and blunted liraglutide-induced weight loss in obese mice (125). Taken together, a growing body of literature suggests that GLP-1RA–induced feeding suppression and weight loss are mediated by multiple GLP-1R–expressing CNS populations and their downstream targets.

Several studies have explored the role of GLP-1R–expressing VANs in GLP-1RA–induced weight loss and appetite suppression and results are mixed. Early studies showed that hindbrain GLP-1R blockade did not attenuate the appetite-suppressing effects of peripheral GLP-1 administration, and that vagotomy partially blocked the acute anorectic effect of peripheral GLP-1RA administration (88, 129, 130). However, reduced food intake in vehicle-treated vagotomized animals confounds interpretation of some of these findings. Chemogenetic activation of GLP-1–releasing EECs or GLP-1R–expressing vagal sensory neurons reduced food intake in overnight fasted and ad libitum–fed mice (50, 55, 131). In agreement, a recent study showed that intra-ileal infusion of GLP-1, or chemo- or optogenetic stimulation of ileal L cells, induced gastric distension via activation of GLP-1R on local intestinofugal enteric neurons and sympathetic ganglion activation, which in turn led to anorexia via a spinal afferent neuron to hypothalamus circuit (53). However, neither ablating GLP-1R–expressing nodose ganglion neurons nor selective GLP-1R knockout from VANs in adult animals reduced liraglutide- or semaglutide-induced weight loss, although GLP-1R knockout in this population modestly impaired dulaglutide-induced weight loss (37, 52, 123). Overall, GLP-1Rs on VANs appear to be dispensable for the majority of GLP-1RAs’ antiobesity effects. Whether the recently described spinal afferent circuit contributes to their efficacy has not yet been evaluated (53).

### Aversive effects.

GLP-1RAs cause GI side effects, including nausea, vomiting, diarrhea, constipation, and abdominal pain in most patients. While these side effects are generally mild and improve over time, they lead to medication discontinuation in roughly 5% of patients (120, 132). In other patients, GI side effects prevent titration to maximum dosing and thus may limit efficacy. GLP-1RA–induced aversion is mediated by GLP-1R in the CNS, as liraglutide-induced conditioned taste avoidance is eliminated in CNS GLP-1R–knockout mice (37). Within the brainstem, GLP-1R knockout from the AP but not NTS prevented GLP-1RA–induced taste avoidance, which likely forms via projections to the parabrachial nucleus. Consistent with this observation, AP GLP-1R neurons were activated by multiple aversive stimuli, and diphtheria toxin–mediated ablation of these neurons prevented the development of conditioned taste aversion in response to their administration (123, 133). Activation of NTS GLP-1R neurons induced nonaversive satiety, likely via projections to the PVH, and this circuit was preferentially activated by nutrients rather than non-nutritive aversive stimuli (123). Previously, others showed that AP lesion reduced baseline bodyweight but did not impact liraglutide-induced weight loss in rats (84, 124), raising the hopeful possibility that aversive anorexia is not necessary for GLP-1RA efficacy. However, recent work indicated that nonaversive satiety is not sufficient for liraglutide-induced weight loss in mice. Specifically, restoration of GLP-1R to NTS neurons in a null background did not rescue chronic GLP-1RA–induced weight loss, whereas restoration of GLP-1R in the AP reinstated both drug-induced aversion and weight loss (134). Thus, the acute and chronic effects of GLP-1RA may depend on different neural substrates, and it remains unclear whether selectively targeting NTS versus AP GLP-1R neurons would be a viable approach to improve GLP-1RA tolerability while preserving efficacy.

GLP-1R–expressing vagal afferent neurons likely do not contribute to GLP-1RA–induced aversion, as GLP-1R knockout from this population did not attenuate the acute aversive effect of liraglutide (37), and optogenetic stimulation of GLP-1R–expressing VAN terminals in the NTS did not cause place avoidance in a real-time, closed-loop assay (50). However, optogenetic stimulation of GLP-1R vagal terminals in the NTS did induce a conditioned flavor avoidance, so this population may be able to encode avoidance under certain conditions (55).

**Figure 2:**
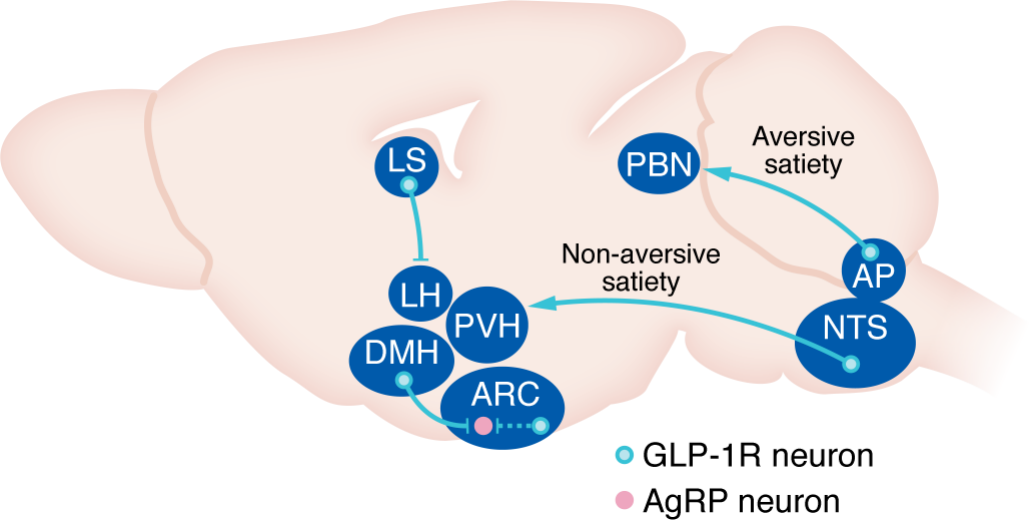
Proposed sites of GLP-1RA action in the brain. Central GLP-1 signaling underlies the weight loss effects of GLP-1RAs. The dashed line indicates a known projection from TRH-/GLP-1R–expressing ARC neurons to AgRP neurons. However, it remains unknown whether GLP-1R knockout from ARC TRH-/GLP-1R–expressing neurons impacts GLP-1RA–induced appetite suppression or weight loss.

Proposed mechanisms underlying GLP-1RA–induced weight loss and aversion are summarized in Figure 2.

## Conclusions, emerging research directions, and critical gaps

Over a decade of investigation using modern neuroscience and genetic approaches has greatly advanced our understanding of the gut-brain mechanisms through which endogenous and pharmacologic GLP-1R activation modulates energy balance and food intake. It is important to acknowledge that this work has been carried out in rodents, and the degree to which specific circuit effects translate to humans has not been determined. Beyond the remaining uncertainties discussed above, emerging clinical data have generated additional questions that are the subject of ongoing and future work.

First, multiple-agonist therapies, including the FDA-approved GLP-1/GIP receptor coagonist tirzepatide, are more effective at promoting weight loss than GLP-1R monoagonists, including semaglutide (135–137). Understanding how glucose-dependent insulinotropic polypeptide (GIP), glucagon, amylin, and other gut hormones contribute to appetite suppression and energy balance using approaches similar to those described above has become an area of rapid growth (138–140).

Second, approximately 10%–15% of patients lose little or no weight on semaglutide, and patients with comorbid type 2 diabetes have less robust weight loss than patients without diabetes (120, 141). The mechanisms underlying response heterogeneity are not understood, and given that GLP-1RA–induced weight loss is centrally mediated the answers likely lie in the brain. A combination of clinical and basic approaches is needed to test this hypothesis and dissect the underlying neural and molecular mediators to enable the development of treatments with efficacy in GLP-1RA nonresponders.

Third, there is mounting evidence that GLP-1RA therapy in patients with obesity and/or type 2 diabetes has cardiovascular (142, 143), renal (144), hepatic (145), and mortality benefits that are partially independent of weight loss (146). These are likely mediated in large part by the pleiotropic antiinflammatory effects of GLP-1RAs (147). In particular, recent, compelling evidence indicates that GLP-1Rs in the CNS are critical for GLP-1RA–mediated reductions in systemic inflammation in a mouse model of sepsis (148). Understanding brain-to-body mechanisms that prevent or reverse inflammation without immunosuppression has implications far beyond the type 2 diabetes and obesity fields and is an area of intense research focus.

Fourth, non-GI side effects are of concern to many patients and merit additional study with respect to their prevalence in at-risk populations and mechanisms. Chief among these is lean mass loss and a risk of sarcopenia, particularly in older patients and those with comorbid type 2 diabetes (149), though it is important to note that physical function improves despite this loss in many patients on GLP-1RAs (150). Pharmacologic approaches to preserve lean mass during GLP-1RA therapy are being actively investigated (151).

Finally, weight regain after medication cessation is an important consideration, as semaglutide is now approved to treat obesity in patients as young as 12 years old (152), and liraglutide efficacy has been evaluated in children as young as 6 years old (153, 154). A major focus in obesity research is to delineate molecular mechanisms and neural circuits required for weight loss versus preventing weight regain, which are likely distinct processes (155, 156). The reason that lifestyle modification alone usually does not lead to sustained weight loss is that negative energy balance results in adaptive responses, including reduced metabolic rate and increased food intake (157). In contrast, patients taking semaglutide do not experience increased hunger and food intake even after meaningful weight loss. One intriguing hypothesis is that AgRP neurons, which are activated by prolonged fasting and necessary for adequate feeding response to negative energy balance in rodents, and which are inhibited by GLP-1RAs (68, 76, 93, 158, 159), could represent a key neural target for weight loss maintenance (160). This and other hypotheses require testing, as the development of specific therapies for weight loss maintenance could prevent the need for decades-long exposure to GLP-1RA therapy.

GLP-1RAs, and more recently the FDA-approved GLP-1R/GIP receptor dual agonist tirzepatide, induce weight loss and improve metabolic outcomes through a distributed system of GLP-1Rs across the gut-brain axis and have dramatically raised the bar for the development of future antiobesity therapies. However, the pace of research in the field, the rapid expansion of neuroscience tools for circuit dissection in preclinical models, and an impressive pipeline of investigational therapies ensure that the next decade will lead to further advances in the medical management of obesity.

## Version 1 01/16/2026

Electronic publication
